# Overexpressed *vs* mutated *Kras* in murine fibroblasts: a molecular phenotyping study

**DOI:** 10.1038/sj.bjc.6604882

**Published:** 2009-02-03

**Authors:** M Horsch, C V Recktenwald, S Schädler, M Hrabé de Angelis, B Seliger, J Beckers

**Affiliations:** 1 Helmholtz Zentrum München – German Research Center for Environmental Health (GmbH), Institute of Experimental Genetics, Ingolstädter Landstraße 1, 85764 Neuherberg, Germany; 2 Martin-Luther-Universität Halle-Wittenberg, Institut für Medizinische Immunologie, Magdeburger Straße 2, 06112 Halle (Saale), Germany; 3 Technical University Munich, Center of Life and Food Sciences, Institute of Experimental Genetics, 85354 Weihenstephan, Germany

**Keywords:** *Kras* mutation, *Kras* overexpression, murine fibroblast cell lines, gene-expression profiling

## Abstract

Ras acts in signalling pathways regulating the activity of multiple cellular functions including cell proliferation, differentiation, and apoptosis. Amino-acid exchanges at position 12, 13, or 61 of the *Kras* gene convert the proto-oncogene into an activated oncogene. Until now, a direct comparison of genome-wide expression profiling studies of *Kras* overexpression and different *Kras* mutant forms in a single assay system has not been carried out. In our study, we focused on the direct comparison of global gene expression effects caused by mutations in codon 12 or 13 of the *Kras* gene and *Kras* overexpression in murine fibroblasts. We determined *Kras* cellular mRNA, Ras protein and activated Ras protein levels. Further, we compared our data to the proteome analysis of the same transfected cell lines. Both overexpression and mutations of *Kras* lead to common altered gene expression patterns. Only two genes, *Lox* and *Col1a1,* were reversely regulated in the *Kras* transfectants. They may contribute to the higher aggressiveness of the *Kras* codon 12 mutation in tumour progression. The functional annotation of differentially expressed genes revealed a high frequency of proteins involved in tumour growth and angiogenesis. These data further support the important role of these genes in tumour-associated angiogenesis.

The Ras gene family (*Hras*, *Nras, Kras4A, and 4B*) encodes small intracellular, membrane-associated proteins, which are regulated by the GDP/GTP cycle. Switching between the active GTP-bound and the inactive GDP-bound state is regulated by GTPase-activating proteins (Marshall, 1995) and guanine nucleotide exchange factor proteins (Downward, 1996). Activated Ras stimulates a cascade of serine/threonine kinases that control diverse biological processes including cell proliferation, differentiation, or apoptosis. The *Kras* proto-oncogene becomes oncogenic by single point mutations in codon 12 or 13, which locks the mutated *Kras* gene product in the GTP-bound activated state (Barbacid, 1987). These mutant forms of Ras transduce signals that result in oncogenic transformation, angiogenesis, invasion, and metastasis by downstream pathways (Olson and Marais, 2000). Frequently, activated *Kras* oncogenes were identified in human bladder, lung, breast, and kidney cancer (Fujita *et al*, 1988; Solana *et al*, 1992; Yuasa *et al*, 1994; Orntoft and Wolf, 1998) as well as in carcinoma of the colon (Capon *et al*, 1983) and in acute myeloid leukaemia (Neubauer *et al*, 1994). In addition to the Ras mutants, the generation of multiple *Kras* gene copies has been detected in murine mammary tumours.

Despite the prevalent role of *Kras* mutations in tumorigenesis, only a few studies have assessed genome-wide transcriptional changes in *Kras*-transfected cell lines. Transcript expression analyses have been performed comparing normal rat embryo fibroblasts and cells transformed by mutant *Hras*, *Kras,* and *Nras* (Zuber *et al*, 2000). Neoplastic transformation driven by the mutated *Kras* oncogene was analysed by expression profiling of rat ovarian epithelial cells (Tchernitsa *et al*, 2004). Differentially regulated genes in response to an activated mutation in *Kras* or *Hras* were analysed in a colon adenoma cell line, employing microarray technology (Roberts *et al*, 2006). Transcriptome analysis described changes in gene expression levels of embryonic mouse fibroblasts carrying a mutation in codon 12 of *Kras* (Vasseur *et al*, 2003). These studies showed that genes regulated by the activated forms of Ras involve cellular processes associated with tumorigenesis.

Clinical studies have suggested that tumour cells carrying *Kras* codon 13 mutations are less aggressive than those with codon 12 mutations (Bazan *et al*, 2002). Different levels of aggressiveness in the transforming phenotype induced by mutations in *Kras* codon 12 or 13 and the overexpression of the *Kras* proto-oncogene in transfected NIH3T3 cells were also described (Guerrero *et al*, 2000). These results suggest that despite many similarities in their oncogenic capacity, the different mutant forms and Ras overexpression may activate also distinct downstream targets that are responsible for the different oncogenic capacities.

So far, a direct comparison of genome-wide transcript profiles of different *Kras* mutant forms and *Kras* overexpression in a single assay system has not been performed. Because of these reasons we used genome-wide expression profiling analysis to investigate differences among samples of murine fibroblasts carrying *Kras* codon 12 or 13 mutations and cells that constitutively overexpress the wild-type (wt) *Kras* gene. In wt *Kras,* the codons at position 12 and 13 encode two adjacent glycines located in the GDP/GTP-binding site of Ras. The G12V and G13D mutations lead to the constitutive binding of GTP and as a consequence to a permanent activation of the signal transduction cascade. We analysed *Kras* NIH3T3 transfectants carrying mutations at codon 12 (Kras^G12V^) or 13 (Kras^G13D^) or overexpress wt *Kras* (Kras^oe^). The non-transfected parental (NIH3T3) and mock-transfected cells (Kras^mock^) served as controls. The total amounts of *Kras* cellular RNA, Ras, and active Ras proteins were measured in all cell lines. Hierarchical clustering of differentially expressed genes was used to compare the gene expression patterns of the distinct transfected cell lines. Furthermore, we analysed the functional classification of the regulated genes and their role in distinct carcinogenic processes.

## Materials and methods

### *Kras*-transfected cell lines

Wildtype and mutated *Kras* genes were amplified by RT–PCR from human cell lines and cloned into the pIRES*hyg* expression vector of murine NIH3T3 fibroblast as recently described (Recktenwald *et al*, 2007). Total RNA from transfecants and non-transfected cell lines was obtained according to the manufacturer's protocols using RNeasy Midi kits (Qiagen, Hilden, Germany). The RNA concentration was calculated from OD_260/280_ readings and 2 *μ*g RNA aliquots were run on formaldehyde agarose gels to check for RNA integrity.

### DNA microarrays

Glass cDNA chips were produced as recently described (Horsch *et al*, 2008). A full description of the approximate 21 000 probes on the microarray is available in the GEO database under GPL3697. The expression data of *Kras*-transfected NIH3T3 cell lines have been submitted to the GEO database (GSE8372).

Four independent dual colour hybridisations including two dye swap experiments were performed with RNA from each of the four transfected cell lines (in total *n*=16) using non-transfected cells as reference. All experiments were performed according to a modified TIGR protocol (Horsch *et al*, 2008). Slides were scanned with a GenePix 4000A microarray scanner and the images analysed with the GenePix Pro6.1 image processing software (Molecular Devices, CA, USA).

### Analysis of gene expression levels

Statistical analyses were performed with TIGR Microarray Data Analysis System (TM4) including MIDAS (Microarray Data Analysis System; Quackenbush, 2002) for normalisation and SAM (significant analysis of microarrays; Tusher *et al*, 2001) for identification of genes with significant differential regulation. Cluster analyses were employed using HCL (hierarchical cluster analyses; Eisen *et al*, 1998).

Expression data were processed (MIDAS) applying a total intensity normalisation, and low-quality array elements were eliminated by several filtering methods, such as background checking for both channels with a signal/noise threshold of 2.0, one bad tolerance policy parameter, and a flip dye consistency check. First, a multiclass SAM analysis for the identification of significant gene regulation in Kras^oe^, Kras^G12V^, and Kras^G13D^ transfectants was performed. Therefore, three groups of experiments were specified: Four chip hybridisations of Kras^oe^-, Kras^G12V^-, and Kras^G13D^-transfected cell lines built a separate group. Genes were considered as significant if they were differentially regulated in at least two of the three specified groups. Second, significantly regulated genes in the Kras^mock^ transfectants were identified by one class analysis. The percentage of genes identified by chance is the false discovery rate (FDR), which was estimated by calculating 1000 permutations. For hierarchical clustering of expression profiles, the average-linkage method was applied. As distance metric the Euclidean distance was chosen.

### *In silico* pathway analysis

For *in silico* analysis of differentially expressed genes, EASE, a module of the DAVID database (Dennis *et al*, 2003) assigning genes to gene ontology (GO) functional categories, was employed. EASE analysis, including a Bonferroni multiplicity correlation, evaluated the set of differentially expressed genes for overrepresentation of two categories of GO terms: biological processes and molecular functions.

### Real-time quantitative (qRT) PCR

Ten differentially expressed genes (*Col6a1*, *Fapb5*, *Ftl1, H3f3b, Lox, Prss23, S100a11, Sparc, Sqstm1, and Vim*) as well as the expression levels of *Kras* were assessed by qRT–PCR performed in a 7700 SDS thermal cycler using the SYBR Green I system (Applied Biosystems, Darmstadt, Germany). Two target-specific primer pairs were designed for each selected gene using OligoPerfect™ Designer (Invitrogen, Karlsruhe, Germany) and purchased from Sigma (Taufkirchen, Germany). Duplicate crossing points per marker were averaged per independent experiment for both primer pairs (*n*=16, including two cDNA dilutions). Values were normalised to levels of *β*-actin.

### Analysis of total and activated Ras expression levels

Expression levels of active GTP-bound Ras as well as total Ras were determined as described previously (Recktenwald *et al*, 2007). Activated Ras molecules were isolated with a selective pull-down assay with glutathione S-transferase-Raf1–Ras-binding domain fusion protein (GST-Raf1–RBD) using the EZ-Detect™-Ras-Activation Kit (Perbio, Bonn, Germany). Cells were harvested, lysed, and incubated with GST-Raf1-RBD and one swell gel glutathione disc. After three washing steps with lysis buffer, GTP-bound Ras was eluted with SDS sample buffer (Laemmli UK 1970) and boiled. Equal protein amounts were separated on 12% SDS polyacrlyamide gels and transferred onto nitro cellulose membranes. Ras molecules were visualised by incubation of the membranes with anti-Ras primary antibody followed by incubating with a horse-raddish peroxidase-coupled antimouse secondary antibody. The complexes were detected using the enhanced chemiluminescence Kit (Perbio).

## Results

### Morphological changes of the *Kras* transfectants

As recently described (Recktenwald *et al*, 2007), the morphology of *Kras* transfectants was characterised by a more spindle-like shape and appeared less flat as parental and mock control cells. Furthermore, the *Kras* transfectants exhibit long cell extensions suggesting the development of filo- and/or lamellipodiae. These morphological changes are most prominent in *Kras* mutants and weaker in the Kras^oe^ transfectants. In addition, all *Kras* transfectants show an increased cell growth as indicated by reduced doubling times (data not shown).

### *Kras* transcript and protein expression levels

**Figure 1:**
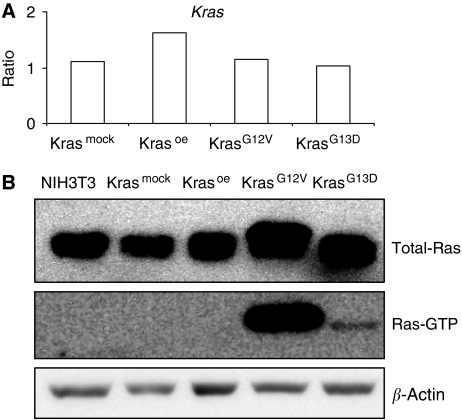
*Kras* transcript and protein expression levels. (**A**) *Kras* expression levels were measured by qRT–PCR. The results are given as the linear ratio of expression levels relative to the expression level of *Kras* expression in the non-transfected NIH3T3 cells. Numbers on the *Y* axis show the fold changes of gene expression levels. (**B**) Quantification of total Ras and activated Ras molecules by western blotting. *β*-actin served as loading control.

We measured the levels of *Kras* gene expression in all cell lines investigated by qRT–PCR. No changes in the total amount of *Kras* in Kras^mock^, Kras^G12V^, and Kras^G13D^ cell lines compared with the non-transfected cell lines were identified (Figure 1A). The overexpression of *Kras* was only detected in Kras^oe^ transfectants. Additionally, for the determination of the Ras protein expression, the activation status as well as the total cellular Ras expression of the different Ras oncoproteins was analysed by western blot analysis. Kras^G12V^ and Kras^G13D^ cells showed similar increased levels of total cellular Ras proteins when compared with the parental- (NIH3T3) and mock-transfected cell lines (Figure 1B). Furthermore, activated GTP-bound Ras was only detectable in these two cell lines (Figure 1B). Although the level of *Kras* transcript was not influenced by the mutations, the protein levels and its activity were increased in Kras^G12V^ and Kras^G13D^ cells. *Kras* overexpression on transcript level had no influence on cellular protein or active Ras protein level.

### Comparative analysis of gene expression patterns

In this study, a comparison of global gene expression effects caused by mutations in codon 12 (Kras^G12V^) or 13 (Kras^G13D^) of the *Kras* proto-oncogene and *Kras* overexpression (Kras^oe^) in murine fibroblasts was performed. A multiclass SAM analysis identified 61 genes significantly regulated in at least two out of the three Kras^oe^, Kras^G12V^, and Kras^G13D^ transfectants compared with non-transfected parental cells. Significance was assigned using an FDR threshold of <0.05 in conjunction with a ratio threshold of >1.5. In all, 17 out of 34 genes significantly regulated in Kras^mock^ transfectants were also identified as differentially expressed in the three experimental cell lines (Kras^G12V^, Kras^G13D^, and Kras^oe^). On the basis of the rational that the altered expression level of these 17 genes was most likely caused by the transfection procedure itself, these genes were excluded from the subsequent hierarchical clustering and GO term analyses.

**Figure 2:**
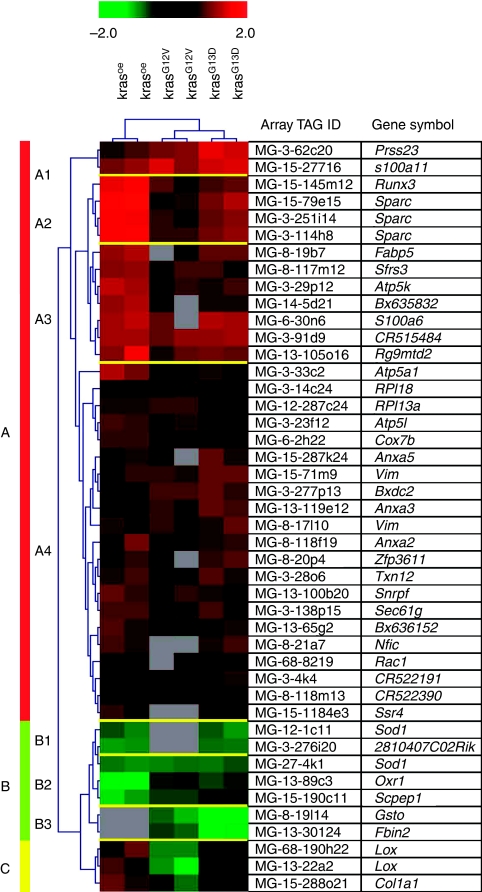
Hierarchical cluster analysis. Hierarchical cluster analysis was performed using gene expression data from 44 probes significantly differentially expressed in at least two out of three transfected NIH3T3 cell lines. For each gene, red indicates higher expression relative to the control and green indicates lower gene expression. Grey boxes represent genes with expression levels below detection limits. Several subgroups of genes with similar expression patterns are colour coded to the left of the heat plots.

Hierarchical cluster analysis was applied on the remaining 44 genes differentially expressed in at least two out of three experimental *Kras* transfectants, but not in the Kras^mock^ cell line. The cluster algorithm classified the selected genes into three groups based on the similarities in expression patterns across the transfectants (Figure 2): the first group comprises upregulated genes (Figure 2, indicated as ‘A’), the second group comprises the downregulated genes (Figure 2, indicated as ‘B’) in the Kras^oe^-, Kras^G12V^-, and/or Kras^G13D^-transfected cell lines. The genes of the subgroup C were upregulated in Kras^oe^, downregulated in Kras^G12V^, and showed no regulation in Kras^G13D^ (Figure 2, indicated as ‘C’). For *Lox*, both non-overlapping sequences represent similar values of transcriptional changes compared with the reference cells. Increased expression levels of 1.8/1.9-fold changes for *Lox/Col1a1* were found in Kras^oe^. In Kras^G12V^, *Lox/Col1a1* was downregulated (2.1/1.6-fold changes). Ratios of −1.07 for *Lox* and 1.13 for *Col1a1* in Kras^G13D^ indicate no significant differences between this transfected cell line and the reference. These data indicate that the overexpression of *Kras* (Kras^oe^) influences the regulation of genes similarly to the regulation induced by the mutations in codon 12 or 13 of *Kras*. Yet, cluster analysis revealed more similarities between gene expression patterns of Kras^G12V^ and Kras^G13D^ transfectants (Figure 2) when compared with Kras^oe^ cells.

**Figure 3:**
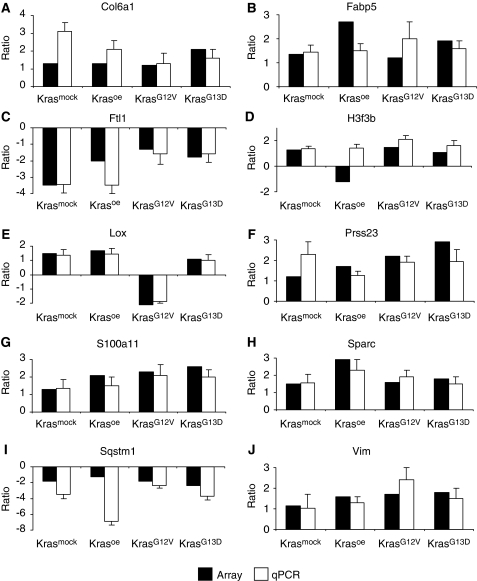
Validation of microarray experiments. qRT–PCR from 10 regulated genes in the four transfected cell lines compared with microarray data. Results are illustrated as ratios of expression relative to the expression of the reference (non-transfected NIH3T3 cells). Black columns represent the mean linear ratio of four microarray experiments, and white columns the mean linear ratios of qRT–PCR. Numbers on the *Y* axis show the fold changes of gene expression levels. Data represented are : (**A**) *Col6a1*, (**B**) *Fapb5*, (**C**) *Ftl1,* (**D**) *H3f3b,* (**E**) *Lox,* (**F**) *Prss23,* (**G**) *S100a11,* (**H**) *Sparc,* (**I**) *Sqstm1,* and (**J**) *Vim*.

Changes in the expression levels of 10 genes (*Col6a1*, *Fabp5*, *Ftl1*, *H3f3b*, *Lox*, *Prss23*, *S100a11*, *Sparc*, *Sqstm1*, and *Vim*) identified as regulated in the microarray experiments were validated by qRT–PCR. With the exception of *H3f3b,* the tendency in terms of up- and downregulation was confirmed in all four cell lines (Figure 3).

### Functional classification of regulated genes

To analyse whether specific functional annotations were overrepresented among the regulated genes, EASE was used to classify the genes for two categories of GO terms: biological processes and molecular functions. The overrepresentation of nine molecular functions was detected (Table 1A): for example, catalytic and structural molecule activity or binding of calcium ions, nucleic acids, lipids, or proteins. GO term analysis of biological processes identified cell growth, development, nucleic acid metabolism, cell proliferation, and protein metabolism as over-represented (Table 1B). Thus, the upregulation of eight calcium-binding proteins suggests that altered intracellular signalling might be associated with constitutive *Kras* activation. Additionally, several genes functionally associated with cell growth, proliferation, development, and structural molecule activity indicate pathways involved in *Kras*-mediated tumour development and metastasis.

## Discussion

In this study, we focused specifically on the direct comparison of the global gene expression effects caused by mutations in codon 12 or 13 of the *Kras* proto-oncogene and *Kras* overexpression. In general, the significantly differentially expressed genes followed the same tendency of regulation in the three transfected cell lines. Yet, cluster analysis revealed more similarities between gene expression patterns of Kras^G12V^ and Kras^G13D^ transfectants compared with Kras^oe^ cells. The level of cellular RNA was only elevated in Kras^oe^, whereas the amount of activated Ras molecules was increased in Kras^G12V^ and Kras^G13D^ cells. The differences in transcript expression patterns among the transfectants might be due to the mutation-mediated constitutive activation of *Kras*. In contrast, the overexpressed form of *Kras* still can switch from the activated to the non-activated state (Wittinghofer, 1998).

There is clear evidence that the activation of the Raf/MAPK pathway is sufficient for oncogenic transformation mediated by Ras (Ellis and Clark, 2000). The expression of wt and mutant *Kras* alleles caused constitutive activation of this pathway as indicated by the phosphorylation status of *Raf*, *Erk1,* and *Erk2* (Recktenwald *et al*, 2007). Our microarray experiments detected no significant changes in gene expression levels of MAP kinases. However, for the overexpressed genes, *Rac1* and *Sparc* regulatory effects in the MAP kinase pathways has been described (Frost *et al*, 1997; Kato *et al*, 2001).

Proteome analysis of the transfected cell lines selected differentially expressed proteins at least 2-fold up- or downregulated compared with NIH3T3 cells (Recktenwald *et al*, 2007). Up to 52 differentially expressed proteins were detected in the various *Kras* transfectants. Our expression profiling analysis identified 44 differentially expressed genes in at least two out of three transfectants (Kras^oe^, Kras^G12V^, and Kras^G13D^). In general, the overlapping transcripts and the proteins followed the same tendency of regulation in the different cell lines. Similar regulation in both transcriptome and proteome studies were found for *Anxa5*, *S100a11*, and *Fapb5* in Kras^G12V^-transfectants as well as for *Sod1* in Kras^oe^. Furthermore, the genes *Lox* and *Col1a1,* and the protein Hsp86 were reversely regulated between the *Kras* transfectants. However, a few proteins (eg, Anxa2) showed a reverse regulation compared with their transcripts. This comparison between differentially expressed proteins and transcripts suggests that changes at the protein level were associated in some cases with a corresponding transcriptional regulation.

Several of the significantly regulated genes identified in our study were annotated with cellular functions such as cell growth (eg, *Atp5a1, Fabp5*, *Runx*, and *S100a6*) and cell death (eg, *S100a11*), angiogenesis (eg, *Anxa2* and *Zfp36l1*), tumorigenesis (eg, *Fabp5*, *Prss23*, and *Runx*), and metastasis (eg, *Lox* and *Sparc*). Genes with very similar functional annotations were also regulated in expression studies of Kras^G12V^-transformed rat embryonic fibroblast (Tchernitsa *et al*, 2004) in tumours derived from Kras^G12V^ mouse embryonic fibroblasts (Vasseur *et al*, 2005) and in a human colon adenoma cell line constitutive active *Kras* due to a mutation in codon 12 (Roberts *et al*, 2006). These data suggest that independent of the cellular system and species background (mouse, rat, or human), the G12V mutation influences the regulation of the same cellular processes.

Potential role of the regulated genes in tumour development and progression were also identified by GO analysis. The genes under the over-represented terms on structural molecular activity, cell growth, and cell proliferation include several genes directly annotated with tumorigenesis. For example, *Runx3* was described as a tumour suppressor in gastric carcinogenesis (Peng *et al*, 2008). Significant overexpression of transcript *Zfp36l1* was found in lymph node and breast carcinomas (Abba *et al*, 2007), and increased *Fabp5* expression induces metastasis in human prostate carcinomas (Morgan *et al*, 2008). Through such functional roles of genes differentially expressed by constitutive *Kras* activation or its overexpression, *Kras* potentially influence tumour progression.

Angiogenesis is a cellular function whereby solid tumours recruit their own blood supply. In microarray studies, it was hypothesised that fibroblast secrete molecules that both promote and inhibit angiogenesis (Pollina *et al*, 2008). *Lox*, *Sparc*, and *Sod1* genes with copper-binding capacity have been associated with angiogenesis (Lane *et al*, 1994; Juarez *et al*, 2006; Shieh *et al*, 2007). Those genes identified in our study to be associated with angiogenesis indicate pathways involved in *Kras*-mediated tumour development and metastasis.

Different levels of aggressiveness in the transforming phenotype induced by mutations in *Kras* codons 12 or 13, and the overexpression of the *Kras* proto-oncogene in transfected tumour cells have been described (Guerrero *et al*, 2000; Bazan *et al*, 2002). In our study, activated Ras was stronger increased in Kras^G12V^ than Kras^G13D^ cells suggesting a diminished protein stability of the Kras^G13D^ oncoprotein. It would be speculated that due to the possible switch from the activated to the non-activated state of overexpressed wt *Kras*, this overexpression has no influence on the amount of activated Ras molecules. Thus, despite many similarities in their oncogenic capacity the different mutant forms and overexpression of *Kras* may lead to a modulated activation of downstream targets that are responsible for these distinct oncogenic capacities. For example, the G12V mutation was more prevalent in metastatic human colorectal carcinoma than the codon 13 mutation (Al-Mulla *et al*, 1998).

The two genes, *Lox* and *Col1a1,* exhibiting either a reverse or non-regulation in the various transfectants may contribute to the different malignant phenotype. Despite the well-known physiological activity of *Lox*, its role in oncogenesis is quite controversially discussed. Elevated expression levels of *Lox* were detected in hypoxic human tumour cells (Erler *et al*, 2006) and in invasive/metastatic human breast cancer cell lines (Payne *et al*, 2005). However, reduced *Lox* levels were also observed in many cancers and cancer-derived cell lines (Csiszar *et al*, 2002; Kaneda *et al*, 2004). Additionally, *Lox* expression could inhibit the transforming activity of the Ras oncogene in NIH3T3 fibroblasts (Min *et al*, 2007) suggesting a possible role of *Lox* as tumour suppressor (Di Donato *et al*, 1997). Paradoxically, *Lox* expression is associated with both tumour suppression and tumour progression. Its role in tumorigenesis seems dependent on cellular location, cell type, and transformation status (Erler *et al*, 2006). The lower expression of *Lox* in the Kras^G12V^-induced tumours may contribute to the higher aggressiveness of this mutation. In contrast, the upregulation of *Lox* in Kras^oe^ transfectants could be interpreted as an important inhibitory factor of the transforming activity of *Kras*.

The downregulation of type I collagen gene (*Col1a1*) is a common feature of Ras transformation (Thomas *et al*, 2005). However, the overexpression of wt Ras itself is apparently not sufficient to reduce the expression of *Col1a1* (Slack *et al*, 1992). Decreased expression levels of *Col1a1* were identified only in Kras^G12V^ transfectants showing the highest Ras activity. Furthermore, no effects of wt Ras overexpression in rat fibroblasts on *Col1a1* mRNA level were found (Slack *et al*, 1992), whereas the gene was upregulated in Kras^oe^ cells. We conclude that the overexpression of *Col1a1* may suppress the transformed phenotype, whereas the downregulation of *Col1a1* mediated by the codon 12 *Kras* mutation contributes to the neoplastic phenotype with the ability of tumour cells to metastasise.

Identification and characterisation of genes differentially regulated by *Kras* overexpression and different *Kras* mutant forms should shed light on the understanding of how this oncogene regulates cell transformation and tumorigenesis.

## Figures and Tables

**Table 1:** Gene ontology

**GO term**	**Gene symbol**
*(A) Molecular functions*	
Calcium–ion binding	*Anxa2, Anxa3, Anxa5, Fbln2, S100A11, S100A6, Sparc, Ssr4*
Nucleic acid binding	*Nfic, RPl18, Runx3, Sfrs3, Snrpf, Zfp36l1*
Catalytic activity	*Anxa3, Atp5a1, Cox7b, Lox, Rac1, Sod1*
Transporter activity	*Atp5a1, Atp5 l, Cox7b, Fabp5, Sec61G*
Structural molecule activity	*Col1a1, Fbln2, Rpl13a, Rpl18, Vim*
Enzyme regulator activity	*Anxa2, Anxa3, anxa5, S100a6*
Lipid binding	*Anxa2, Anxa3, Anxa5, Fabp5*
Oxidoreductase activity	*Cox7b, Lox, Sod1*
Protein binding	*S100A6, Sparc, Vim*
	
*(B) Biological processes*	
Cell growth	*Atp5a1, Atp5 l, Fabp5, Nfic, Rac1, Runx3, S100a11, S100a6, Sec61 g, Ssr4, Zfp36l1*
Development	*Anxa2, Col1a1, Cox7b, Fabp5, Rac1, S100a6, Sod1, Sparc*
Nucleic acid metabolism	*Atp5a1, Atp5 l, Nfic, Runx3, S100a11, Sfrs3, Snrpf*
Cell proliferation	*Nfic, Ras1, Runx3, S100a11, S100a6, Zfp36l1*
Protein metabolism	*Lox, Rpl13a, Rpl18, Sec61g*
